# Estimating incidence and prevalence rates of chronic diseases using disease modeling

**DOI:** 10.1186/s12963-017-0130-8

**Published:** 2017-04-05

**Authors:** Hendrike C. Boshuizen, Marinus J. J. C. Poos, Marjan van den Akker, Kees van Boven, Joke C. Korevaar, Margot W. M. de Waal, Marion C. J. Biermans, Nancy Hoeymans

**Affiliations:** 1 PO box 1 3720 BA, Bilthoven, The Netherlands; 2 Biometrics, Wageningen University, PO Box 16 6700AA, Wageningen, The Netherlands; 3 Department of Family Medicine, Maastricht University, P.O. Box 616, 6200 MD Maastricht, The Netherlands; 4 Department of Public Health and Primary Care, Academic Center for General Practice, Kapucijnenvoer 33, blok J, PB 7001, 3000 Leuven, KU Belgium; 5 Department of Primary and Community Care, Radboud university medical center, Internal post ELG 117, P.O. Box 9101, 6500 HB Nijmegen, The Netherlands; 6 Netherlands Institute for Health Services Research (NIVEL), Otterstraat 118-124, P.O. Box 1568, 3500 BN Utrecht, The Netherlands; 7 Department of Public Health and Primary Care, Leiden University Medical Center, Postal zone V-0-P, PO-box 9600, 2300 RC Leiden, The Netherlands

**Keywords:** Incidence-prevalence-mortality model, General practitioner, General practice registration, Morbidity, Public health, Burden of disease

## Abstract

**Background:**

Morbidity estimates between different GP registration networks show large, unexplained variations. This research explores the potential of modeling differences between networks in distinguishing new (incident) cases from existing (prevalent) cases in obtaining more reliable estimates.

**Methods:**

Data from five Dutch GP registration networks and data on four chronic diseases (chronic obstructive pulmonary disease [COPD], diabetes, heart failure, and osteoarthritis of the knee) were used. A joint model (DisMod model) was fitted using all information on morbidity (incidence and prevalence) and mortality in each network, including a factor for misclassification of prevalent cases as incident cases.

**Results:**

The observed estimates vary considerably between networks. Using disease modeling including a misclassification term improved the consistency between prevalence and incidence rates, but did not systematically decrease the variation between networks. Osteoarthritis of the knee showed large modeled misclassifications, especially in episode of care-based registries.

**Conclusion:**

Registries that code episodes of care rather than disease generally provide lower estimates of the prevalence of chronic diseases requiring low levels of health care such as osteoarthritis. For other diseases, modeling misclassification rates does not systematically decrease the variation between registration networks. Using disease modeling provides insight in the reliability of estimates.

## Background and goal

For health policy purposes, population health is monitored on a regular basis. An important measure for population health is the morbidity in the population: what are the most important diseases and how are disease patterns changing over time? Registries in general practice are key sources for morbidity estimates, especially if all people are registered in a general practice and the general practitioner (GP) is the gatekeeper of health care. In this case, the population registered in general practices is representative of the whole population outside of long term health care facilities. Furthermore, if the general practitioner acts as a gatekeeper of health care, diagnoses from medical specialists and other health care providers will also be known by the general practitioner. In the Dutch system, both conditions are met. Furthermore, the Netherlands has the fortunate position of having many general practice registration networks. These networks constitute of several general practices, all collecting information on morbidity and health care in a standardized manner [1].

**Fig. 1:**
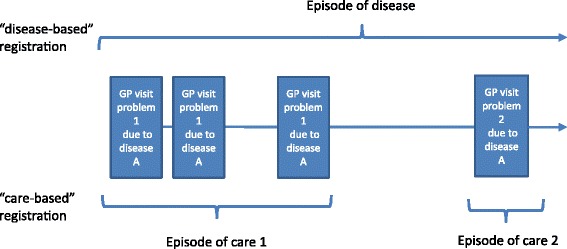
The difference between networks based on episodes of care and episodes of disease

Previous research has shown that morbidity estimates between different general practice registration networks vary considerably [2]. These differences could not be explained by differences in characteristics of the patient population or in the characteristics of the general practice [3, 4]. Most likely, differences in registration procedures contribute to this variation. In particular, the method to distinguish new cases (incidence) from existing cases (prevalence) is expected to have an effect on these estimates. General practice registration networks can count either “episodes of disease” or “episodes of care”. In “episode of care”-based registries, the GP indicates whether the episode is an incident (new) or prevalent one, and for chronic diseases that do not require continuous care, multiple care episodes can be present for the same episode of disease. In “episode of disease”-based registries, only the presence of a (new) disease is counted (Fig. 1). For public health purposes, we want to summarize data from both sources into one measure in order to use all available information. Therefore, we developed a method to enhance the comparability of morbidity estimates from different registries.

The primary aim of this study is to ascertain the consistency of estimates of incidence and prevalence rates of a number of chronic diseases important for public health monitoring. Incidence, prevalence, and mortality are consistent if the prevalence is a credible function of inflow (incidence) and outflow (institutionalization and mortality), taking the possibility of time trends into account. Consistent estimates are needed for chronic disease modeling [5]. Furthermore, consistency improves the reliability of the incidence and prevalence estimates. The secondary aim of this research is to investigate to what extent differences between the networks in the method of distinguishing incident from prevalent cases explain differences in morbidity outcomes between GP registration networks.

## Methods

### Five general practice registration networks

We used data from 2010 from five Dutch general practice registration networks. Although more registration networks exist in the Netherlands [1], these five have been used for population health monitoring [6, 7]. Table 1 shows the full names, abbreviations, and some characteristics of these five networks in 2010. One network has a population representative for the Dutch population. The other networks operate in specific geographical areas in the Netherlands. Networks differ in size, ranging from 14,000 to 270,000 person years and 4–51 general practices.

**Table 1:** General characteristics of five Dutch general practice registration networks in 2010

	LINH	CMR-N	RNUH-LEO	Transition	RNH
Full name	Netherlands Information Network of General Practice	Continuous Morbidity Registration Nijmegen	Registration Network of General Practitioners associated with Leiden University	Transition Project	Registration Network Family Practices
Localization	National	Nijmegen and surrounding area	Leiden and surrounding area	Amstelveen and Franeker	Province of Limburg
Number of person years	270,000	14,000	44,000	14,000	88,000
Number of GPs/practices	85^a^/51	11/4	20/4	8/5	65/22
Source of data	Episode of care	Episode of disease	Episode of disease	Episode of care	Episode of disease

^a^Full time equivalent of GP’s working in the LINH practices

An important difference between the networks is whether “episodes of care” or “episodes of disease” are used as the source of morbidity estimates. An “episode of disease” refers to the *presence* of a disease, while an “episode of care” refers to the *treatment* of a disease [8]. In this latter type of registries, only those diseases are counted for which care was provided. Information on contacts (consultations, visits, phone calls, prescriptions) form the basis for the construction of “care episodes” and with that for the estimates of morbidity rates [9–12]. For example, a patient with known hearing loss who did not contact the GP for this problem in a certain time frame, counts as a prevalent case in “episode of disease”-based registries, but does not count as a prevalent case in “episode of care”-based registries during that time frame.

The networks all use data that are extracted from routine health care systems, filled on a daily basis by the health care providers in general practice. These systems all have roughly similar data-tables, defined in the HIS-reference model (https://www.nhg.org/themas/publicaties/his-referentiemodel). GP practices open a record for each contact with a patient and for each contact with a GP an ICPC code is assigned. That could be a new code, but the contact can also be made part of an existing open episode of disease or care. For chronic diseases, such episodes stay open indefinitely in disease-based networks. For diseases requiring continuous care, the episodes stay open in all networks. New contacts can be assigned to an existing open episode. When the contact refers to multiple health problems, multiple ICPC codes can be entered.

The records from the routine health care systems are transferred to the network coordinating group at fixed times (varying between the networks, but at least once a year). The data transferred also include the number of patients registered with the GP practice, including those that did not contact the GP practice in the period of data capture. Data from different transfer moments can be linked using unique patient numbers, and thus patients can be followed over time. Dates of leaving the practise are not always registered. If a patient is registered at one data transfer date, but not at the next, the patient is assumed to contribute halve the period to the person years in observation.

Changes in the data can be made in the process of routine health care, where GPs remove errors when detected. Data are fixed, however, at the moment of transfer to the network coordinating center. The coordinating centers apply data cleaning, which can include procedures that further link contacts together into episodes.

In an earlier publication, we described these five networks in more detail [1]. Apart from incidence and prevalence rates, the networks also provided data on the number of patients that died. Three of the five networks had information on the number of patients that left the practice because they moved to a nursing home. This information is important, because these patients no longer receive general practice care from their general practitioner and have a larger odds of dying in the next year.

### Incidence and prevalence of four chronic diseases

Four chronic diseases were selected: chronic obstructive pulmonary disease (COPD) (International Classification for Primary Care [ICPC] codes R91 and R95), diabetes (ICPC code T90), heart failure (ICPC code K77), and osteoarthritis of the knee (ICPC code L90). For these diseases, general practice registration networks are the main data source, and these represent a broad spectrum of chronic diseases. Furthermore, these diseases differ in the expected frequencies of visits to the GP. For diabetes, guidelines require general practitioners to see their patients several times each year. On the other extreme, patients once diagnosed with osteoarthritis of the knee do not need to contact their general practitioner very often for this condition.

In this study, estimates of incidence and prevalence rates from networks that use “episodes of care”-based registration methods are obtained by using the information of three calendar years in the registration. Only patients who were registered in GP practice on the first of January 2008 were included in this analysis. To estimate the incidence and prevalence in 2010, we included diagnoses of the specific disease in these patients in the 2 years prior to 2010, and only count a case as an incident case when no episode of care starting before 2010 was observed. Three years were taken as earlier work showed that this is long enough for diseases requiring continuous GP care, while a longer period would imply excluding more patients (those not registered with the GP for that longer period).

For disease modeling, transition rates from healthy to the “with disease” state are needed.

These incidence (density) rates were calculated as the ratio of the number of new cases registered divided by the number of person years free of disease. The latter was estimated from the total number of person years, the number of existing cases of the disease (point prevalence), the number of new cases of the disease (incidence), and the number of persons with the disease that left the general practice due to death or institutionalization. In “episode of disease”-based registries, the point prevalence was calculated as the number of known patients with disease on the first of January divided by the total number of patients in the registration at that moment. For networks based on “episodes of care,” it was calculated as the number of patients seen for the disease in the period of 2008–2010 minus the new, incident patients seen only in 2010, divided by the number of total number of patients registered on the first of January of 2010. The number of patients on the first of January was not provided by all registries, and was therefore estimated as being equal to the number of person years in 2010, implicitly assuming a stable population.

Subsequently, we estimated the incidence and prevalence rates for the whole Dutch population based on the information in all five networks in two different ways. First, the simple mean over the five registries of the age- and sex-standardized prevalence and incidence is calculated. Second, a multi-level model is fitted to the data (with registration network as level, that is, as random intercept), and the age-standardized predicted value from this model is used as estimate. For incidence, a random intercept Poisson model was fitted, and for prevalence a random intercept logistic model was fitted. Third degree polynomials were considered for age and for the interaction of age with sex. We selected the simplest model that *fitted* the data statistically significantly better than all other models (for more detail on this method see [5]).

### Disease modeling

We used disease modeling to check the consistency of the prevalence and incidence figures in each of the networks. For chronic diseases, disease modeling only involves incidence, prevalence, and mortality. By definition, no data on recovery of disease is needed. In the first step of disease modeling, we projected the number of prevalent cases in the network in a hypothetical cohort of newborns, by applying the disease inflow (incidence) and disease outflow (mortality and institutionalization of those with the disease) data of the network to that cohort. This implicitly assumes no important time-trends in incidence and excess mortality (within the period in which the current prevalent cases arose). Also, it assumes that those entering the GP population, and those leaving it for other reasons than death or institutionalization, are similar to those of similar age and sex who stay in the population. We then compared this projected prevalence to the observed prevalence in all age and gender groups.

Second, we fitted a joint model (the DisMod model) using all information on morbidity and mortality in each network to construct the most reliable estimations of prevalence and incidence rates of these four diseases for each network under the assumption of stable incidence and survival probabilities over time. See appendix A for the equations and assumptions of the DisMod model. The model included a misclassification factor that allows for misclassification of a percentage of prevalent cases as incident cases, in order to see whether this might explain the observed lack of prevalent cases compared to incident cases in some situations. We thus assumed that misclassification of incident cases as prevalent cases would be relative rare, and thus can be ignored. We fitted this model by maximum likelihood, separately for each registration. From the fitted model, we calculated the standardized incidence and prevalence rates. For each registration, we generated 1,000 model parameter values from the estimated model parameter vector and its covariance matrix in order to generate the confidence interval. As the numbers of deaths were low in the smaller registrations, institutionalization data were not available in two registrations and moreover, there were clear indications of under registration of deaths in most registrations, the model did not converge reliably for all registrations (see appendix B for the life expectancies as calculated from the mortality data in each registration). Therefore, we decided to use the mortality and institutionalization data from the largest registration (LINH) for all registrations. As can be seen in appendix B, the mortality data from this network where the best calibrated rates in terms of magnitude. The registration-specific standardized incidence and prevalence rates were then combined into an overall standardized incidence or prevalence, using a random-effect meta-analytic model (R package meta, function metagen) on the logarithm of the standardized incidence or prevalence and its standard error.

## Results

The mean incidence of diabetes is 4.6 per 1,000 (simple mean), and this figure varies from 3.7 in the CMR-N registration to 5.4 in the Transition registration. Table 2 shows the incidence rates in all registration networks, for all of the four diseases, as well as the two different estimates of the mean morbidity. The highest estimate of incidence is 1.5 (diabetes) to 2.7 (heart failure) times higher than the lowest estimate. The fitted means are generally a little lower than the simple means. The most important reason is that LINH has lower estimates of incidence while the data from this network determine a larger proportion in the calculation of the mean, because this network includes much more patients compared to the other networks. For prevalence rates, the differences between simple and fitted mean are in the same order of magnitude (see Table 3). Only differences in the prevalence of osteoarthritis of the knee are larger. These vary between 1.0% in the RNUH-LEO network to 3.9% in the CMR-N network. These differences are reflected in the width of the confidence intervals of the fitted means.

**Table 2:** Incidence (per 1,000) of four diseases for each network, standardized for age and gender in 2010

	Diabetes	COPD	Heart failure	Osteoarthritis of the knee
Observed estimates				
LINH	4.1	2.2	1.8	2.4
CMR-N	3.7	2.6	2.4	3.4
RNUH-LEO	4.7	3.0	2.6	3.5
Transition	5.4	3.9	4.8	4.5
RNH	4.8	4.4	2.2	3.2
Summary estimates				
Simple mean	4.6	3.2	2.8	3.4
Fitted mean (95% CI)	4.5 (4.1 – 4.9)	3.0 (2.3 – 3.8)	2.5 (1.8 – 3.2)	3.2 (2.6 – 3.7)

**Table 3:** Prevalence (per 1,000) of four diseases for each network, standardized for age and gender in 2010

	Diabetes	COPD	Heart failure	Osteoarthritis of the knee
Observed estimates				
LINH	54	26	11	12
CMR-N	48	22	13	39
RNUH-LEO	50	25	11	10
Transition	61	22	18	20
RNH	57	33	8	28
Summary estimates				
Simple mean	54	25	12	22
Fitted mean (95% CI)	54 (50 – 58)	26 (22 – 29)	12 (9 – 15)	19 (11 – 28)

### Disease modeling: examine consistency of incidence, prevalence, and mortality rates

**Fig. 2:**
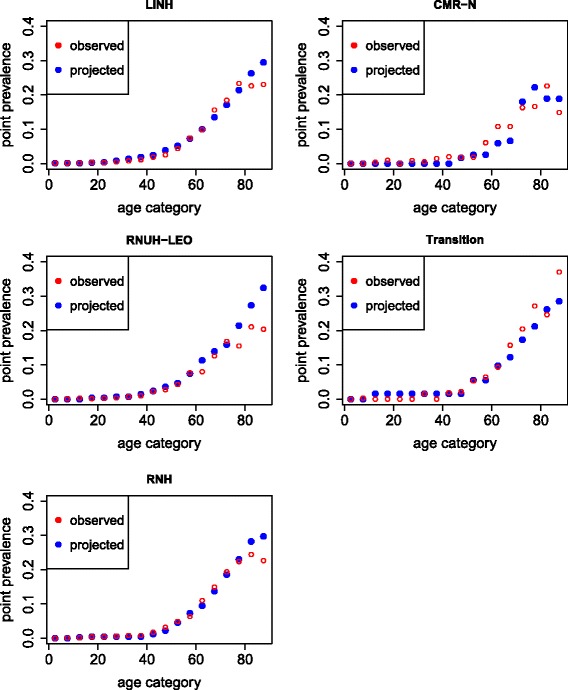
Observed and projected prevalence of diabetes mellitus in women in 2010 by GP network

Figure 2 shows the observed and projected prevalence rates of diabetes in women in the different general practice registration networks by age. In all networks, the projected prevalence rates resembled the observed rate relatively well, meaning that incidence, prevalence and mortality rates were consistent. Only at older ages, the observed prevalence was (a little) lower than expected. This means that the prevalence was expected to be higher, based on the current incidence and mortality rates. For men, the gap between observed and projected prevalence rates was larger than for women (results not shown).

**Fig. 3:**
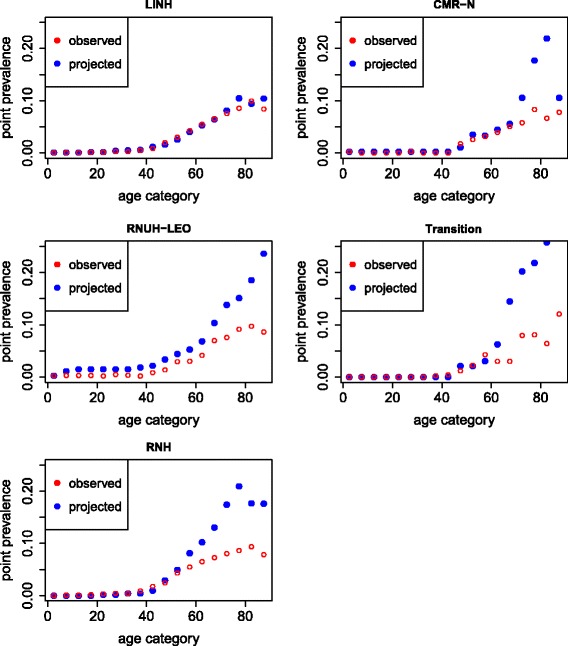
Observed and projected prevalence of COPD in women in 2010 by GP network

The results for heart failure also show incidence, prevalence, and mortality to be rather consistent (see appendix B). For the other two diseases we studied, the consistency was lower. The prevalence rates of COPD as registered by the networks were lower than the projected prevalence rates based on the currently observed incidence and mortality rates. Only in the LINH network were incidence, prevalence, and mortality rates consistent. Figure 3 shows these results for women, but results for men are similar. Results for osteoarthritis show that prevalence rates were rather consistent with incidence rates and mortality for two networks (RNH and CMR-N). The other three networks (Transition, LINH, RNUH-LEO) had observed prevalence rates that were much lower than projected rates (see appendix B).

An important reason for the result that a lower than projected prevalence rate was observed for older ages is that all registration networks underestimate mortality. Appendix C shows that the age-specific life expectancies calculated from the mortality figures as registered by the different networks are much higher than mortality data from Statistics Netherlands. Due to this underestimation of mortality in the networks, the projected prevalence is too high. The reason is that this prevalence is based on incidence (inflow) and mortality and institutionalization (outflow). If mortality is actually higher than registered, the outflow with which we do the calculations is too low, and the subsequent projected prevalence is too high.

### Disease modeling: reliable and consistent estimates of prevalence and incidence

Table 4 shows the fractions of prevalent cases estimated to be misclassified as incident cases according to our model. High misclassification rates were found for osteoarthritis of the knee (with the exception of the CMR-N, a registration that has been in place since 1971, where the misclassification was not statistically significant), while no statistically significant misclassification was present for heart failure. Note that the model assumes that no patients with the disease are missed in the registration. When prevalent cases are missed, this will also result in a higher estimate for the number of misclassified cases, so a high misclassification rate could also indicate that some patients are missed by the registration. When disease modeling takes this misclassification into account, modeled incidence rates are expected to be lower than observed incidence rates. Vice versa, modeled prevalence rates are expected to be higher than observed rates. This effect is expected to be largest for osteoarthritis of the knee, followed by COPD and diabetes, and absent for heart failure.

**Table 4:** Estimated misclassification fraction: percentage of prevalent case that is misclassified as an incident case as estimated by the DISMOD model

	Diabetes	COPD	Heart failure	Osteoarthritis of the knee
LINH males	0^a^	0^a^	0^a^	14.1 (11.3-16.9)
LINH females	0^a^	1.1 (0-2.5)	0^a^	12.4 (10.4-14.2)
CMR_N males	1.5 (0-4.2)	1.4 (0-6.7)	0^a^	4.0 (0-9.0)
CMR_N females	2.8 (0-5.7)	4.7 (0-10.7)	0^a^	0^a^
RNUH-LEO males	3.8 (1.9-5.8)	3.6 (0.1-7.0)	7.2 (0-17.0)	26.4 (17.8-35.0)
RNUH-LEO females	2.7 (0.7-4.6)	4.7 (1.7-7.7)	0^a^	21.6 (15.7-27.6)
Transition males	2.3 (0.7-4.6)	4.4 (0-10.7)	0^a^	17.3 (6.3-28.3)
Transition females	0^a^	9.0 (2.4-15.6)	0^a^	9.1 (3.7-14.4)
RNH males	1.6 (0.5-2.8)	4.8 (2.9-6.7)	3.1 (0-9.1)	6.1 (4.1-8.2)
RNH females	1.4 (0.4-2.5)	6.4(4.7-8.2)	5.0 (0-11.5)	3.4 (2.0-4.9)

^a^No confidence interval calculated because the point estimate is on the boundary of the parameter space

Table 5 shows the incidence rates from the joint model. In a few cases, the confidence intervals around the estimates for individual registrations are wide, indicating that the model does not fit these observed data well. This is an important sign that the incidence and prevalence as registered are not consistent with those expected in a relatively stable population. As expected, incidence was lower in these models as compared to the observed rates, as copied from Table 2. For the diseases with low misclassification rates (diabetes and heart failure), this difference was only moderate. The difference was considerable for osteoarthritis of the knee. In these cases, the estimated overall incidence was less than half of the registered incidence. After applying the DisMod model, the ranking of network incidence rates for osteoarthritis of the knee resembled the ranking of the observed prevalence rates, rather than that of the observed incidence rates. Another effect of the joint modeling is that incidence rates are also based on the observed prevalence rates, which are based on larger numbers than observed incidences rates. Therefore, the confidence intervals around the incidence rates from the joint model are narrower than those from the model on incidence data alone.

**Table 5:** Incidence (per 1,000) as estimated with the DisMod model of four diseases for each network, standardized for age and gender

	Diabetes	COPD	Heart failure	Osteoarthritis of the knee
LINH	4.0 (3.8 – 4.2)	2.1 (1.9 – 2.2)	2.0 (1.9 – 2.2)	0.8 (0.8 – 0.9)
CMR_N	3.3 (3.0 – 3.8)	1.9 (1.7 – 2.4)	2.9 (2.4 – 3.9)	3.0 (2.7 – 3.6)
RNUH-LEO	3.2 (3.0 – 3.6)	2.1 (1.9 – 2.4)	2.5 (2.2 – 2.9)	0.9 (0.8 – 1.2)
Transition	4.8 (4.4 – 5.5)	2.2 (2.0 – 3.2)	6.3 (5.3 – 8.1)	1.5 (1.4 –1640)
RNH	3.9 (3.7 – 4.1)	2.5 (2.3 – 2.7)	2.0 ^a^	1.9 (1.8 – 2.0)
Simple mean over networks	3.8 (3.4 – 4.2)	2.2 (1.9 – 2.4)	2.3 (2.1 – 2.5)	1.3 (0.8 – 2.3)
Fitted mean (table 2)	4.5 (4.1 – 4.9)	3.0 (2.3 – 3.8)	2.5 (1.8 – 3.2)	3.2 (2.6 – 3.7)

^a^Convergence not reliable: no calculation of confidence intervals

Table 6 shows the prevalence rates from the joint model. The estimated prevalence from the DisMod-model was for COPD and osteoarthritis of the knee slightly higher than the observed prevalence, as was expected. The difference, however, was rather small compared to the confidence intervals. For heart failure, where misclassification was estimated to be zero in most cases, the estimated prevalence from the DisMod model was slightly lower than the observed prevalence, but again the difference was small compared to the confidence interval. The prevalence of diabetes was similar for both methods.

**Table 6:** Prevalence (per 1.000) as estimated with the DisMod model, for four diseases for each network, standardized for age and gender

	Diabetes	COPD	Heart failure	Osteoarthritis of the knee
LINH	54 (52 – 55)	26 (25 – 27)	10 (10 – 11)	14 (10 – 14)
CMR-N	48 (45 – 52)	22 (21 – 27)	12 (10 – 15)	39 (31 – 44)
RNUH-LEO	51 (48 – 53)	26 (23 – 27)	11 (10 – 12)	13 (10 – 22)
Transition	61 (57 – 65)	23 (21 – 32)	18 (16 – 21)	22 (18 – 494)
RNH	58 (55 – 59)	34 (32 – 35)	9 ^a^	29 (20 – 30)
Simple mean over networks	54 (51 – 58)	27 (23 – 32)	10 (08 – 12)	22 (13 – 34)
Fitted mean (table 2)	54 (50 – 58)	26 (22 – 29)	12 (09 – 15)	19 (11 – 28)

^a^Convergence not reliable: no calculation of confidence intervals

## Discussion

Prevalence and incidence data from “episode of care” registries based on data that include GP visits from 2 year before baseline yield consistent estimates for diabetes, COPD, and heart failure. Osteoarthritis of the knee, however, could only be consistently estimated from data of a long-running “episode of disease” registration that also registers prevalent diseases of new patients. Using disease modeling including a misclassification term did improve the consistency between prevalence and incidence rates, but did not systematically decrease the variation between networks. This DisMod modeling mostly affected the incidence rates, while prevalence rates did not change importantly. Osteoarthritis of the knee showed a different pattern, as inconsistency between prevalence and incidence rates were large, and are probably not only due to misclassification of incidence as prevalence, but also to missing cases altogether, because prevalent patients with osteoarthritis might not have visited the GP for this disease in a 3 year period. Including misclassification in the disease modeling cannot account for this. Missing cases is to be expected in registries where morbidity is based on “episodes of care,” but also one of the “episodes of disease”-based registrations (RNUH-LEO) yielded large discrepancies between incidence and prevalence. For the other two networks where morbidity is based on “episodes of disease” the consistency was better; that is, observed and projected prevalence matched considerably better. This was especially the case in CRM-N, a very long-running disease episode-based registry.

These results are not unexpected, because osteoarthritis of the knee is a protracted disease, where patients pay fewer visits to their GP (for this specific disease) compared to patients with the other diseases, and visits might be many years apart. The reason is not only that no regular checkups are needed, but also because patients can visit a physical therapist without referral from their general practitioner. Especially if the morbidity registration is based on “episodes of care,” this leads to underestimation of prevalence of disease as well as to misclassifications between incidence and prevalence rates. For instance, in these registrations, osteoarthritis patients, who visit the GP in the year of registration, but did not visit their physician in the 2 years before baseline, will be missed as prevalent cases. These will be counted as incident cases instead, while those not visiting the GP in all 3 years will not be counted at all. For such diseases, a longer period will be needed to obtain reliable estimates of incidence and prevalence.

Differences between the networks are adjusted for age and gender differences of the enlisted population. We did not adjust for other characteristics of the populations, such as socioeconomic status, ethnicity, and degree of urbanization. In an earlier study, we already showed that these characteristics did not explain any of the variance between networks [3]. The same was found for differences between GPs and general practices. Differences in type of practice (solo, duo, or group), mean years of medical experience, and distance to nearest hospital did not explain the variance between the networks [4].

There are further factors in procedures per registration that could influence the incidence and prevalence from GP registrations that we could not study here. For instance, if registrations differ in the way they enter patients that purely received secondary care, making different choice in assigning ICPC codes (due to differences in the clinical definition of the disease or errors in diagnosis) or the frequency in updating earlier diagnoses. Such factors can only be ruled out when strict clinical definitions are applied.

In this study, estimates of incidence and prevalence rates from networks that use “episodes of care”-based registration methods are obtained by using the information of three calendar years in the registration. To estimate the incidence and prevalence of 2010, we included diagnoses of the specific disease in these patients in the 2 years prior to 2010. Until now, only 1 year estimates were used to estimate morbidity in the Netherlands. In this study, we analyzed the misclassifications, and observed that registered incident cases in 2010 regularly had already had episodes in 2008 and 2009 (data not shown). Using data from 2010 only led to predicted prevalence rates that were clearly inconsistent with the observed ones. We concluded therefore that using 1 year data leads to important misclassification in “episode of care”-based registries. Therefore, we used the 3 year data and we also recommend that this is done in future research.

We and others [3, 4] looked at differences between registration network within the same country and found large differences that could not be fully explained. When comparing registration-based data between countries, we expected similar differences in the organization of data collection as between networks in the Netherlands. Moreover, while registration networks in the Netherlands share a health care system and GPs that are trained by and large by the same medical schools, this will not be the case for networks based in different countries. So in the international context confronting prevalence and incidence, data is therefore even more important.

Mortality in general practice was generally underestimated. We did expected mortality to be slightly lower than official statistics, because those with highest mortality rates move to a nursing home. These homes supply medical care for their patients, and so these patients leave the general practitioner and the general practice registration. However, this effect should be similar for all registrations, while we observed clear differences between registrations with regards to the amount of underestimation. However, in case of the LINH the higher mortality compared to other registries might well be an artifact. In this registry, GPs report every three months whether patients are still present, and time of death in those who died was mostly not available. Because of this, the registered cases of death were from a period with unknown length that we assumed to be 1 year, but was on average probably longer. In our models, we also included the patients who moved to an institution. In subsequent research, we advise to use linkage to national mortality data when possible, accounting for privacy legislation.

In order to derive consistent estimates of prevalence, incidence and excess mortality we fitted a joint model to all data. A crucial assumption in this model is that the disease process is stable over time. That is, that incidence and mortality have not changed importantly in recent years (within the duration of the disease). For diabetes in women, this might not be realistic, as the incidence has increased in recent years. Data from the RNH show that the incidence of diabetes doubled in women and tripled in men in the period 1990–2007. Data from CMR-N, showed an increase of 40% and 70% respectively. This implies that past incidence rates were lower than those expected under our model. This might generate a spuriously high misclassification rate. In situations where the disease-related mortality or the incidence has decreased in recent years, the observed prevalence is lower than expected under our model, causing the misclassification rate to be spuriously low. Therefore, the misclassification rates in Table 4 should be interpreted with caution. However, time trends in incidence and mortality can be assumed to be general trends in the Dutch population, and should be the same for all registrations. The clear differences between registrations in the degree in which observed and projected prevalence rates match, indicates that a large part of these differences is due to registration-related misclassification rather than to time trends.

## Conclusion and recommendations

Using disease modeling to compare prevalence with incidence rates is useful for detecting inconsistencies in the data. Comparing observed and modeled data provides insight into the reliability of the data. We applied this for the Netherlands, but this will be useful too if data are compared between registries from different countries. Data from 1-year “episode of care”-based data turned out to be unreliable, while those based on a 3 year period are sustainable. For osteoarthritis, this was only the case for data based on a long-running “episodes of disease” registration. For such chronic diseases for which there is no regular contact with a physician, an “episodes of disease” registry is essential; otherwise, too many prevalent cases are missed to build a reliable morbidity register. Disease modeling can be improved further by using more reliable mortality data.

## Figures and Tables

**Fig. 4:**
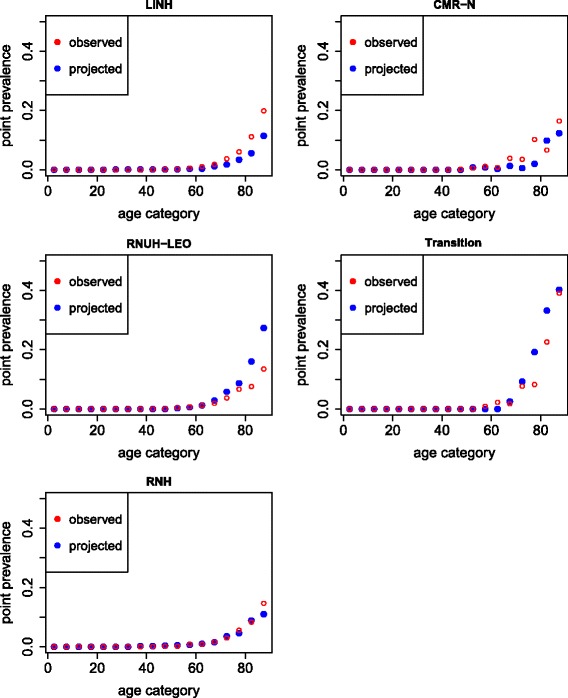
Observed versus projected prevalence of heart failure in men in 2010 by GP network

**Fig. 5:**
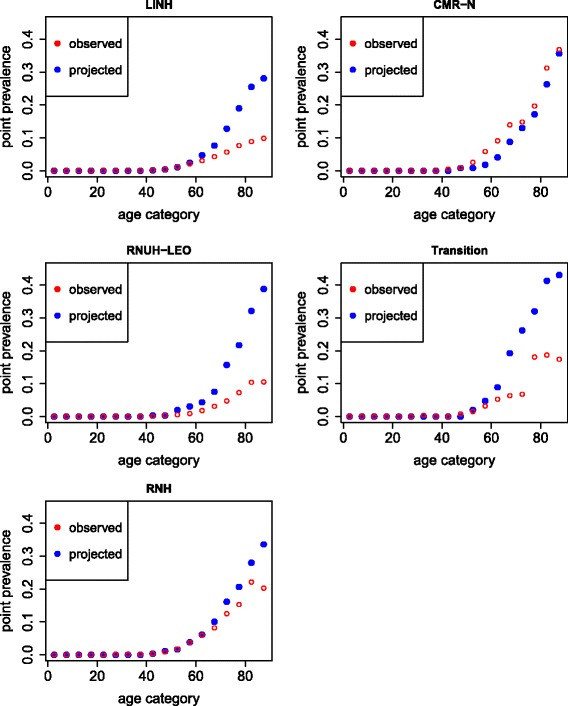
Observed versus projected prevalence of osteoarthritis of the knee in women in 2010 by GP network

**Fig. 6:**
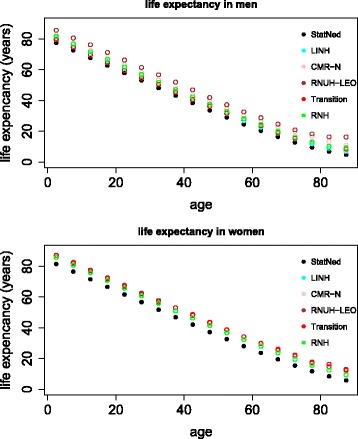
Life expectancy in men (top) and women (bottom), as calculated on mortality data from the general practice registration networks and from Statistics Netherlands

## Appendix A

### DisMod model

The DisMod model comprised of the following system of equations:

$$ \begin{array}{l} i(a)={\alpha}_i+{\beta}_1 a+{\beta}_2{a}^2+{\beta}_3{a}^3\\ {} m(a)={\alpha}_m+{\gamma}_1 a+{\gamma}_2{a}^2+{\gamma}_3{a}^3\\ {} \log \left( RR(a)\right)={\alpha}_{RR}+{\delta}_1 a+{\delta}_2{a}^2+{\delta}_3{a}^3\\ {} em(a)= m(a)\left( RR(a)-1\right)\\ {} p(a)=\frac{\left(1- p\left( a-1\right)\left)* tr(a)+ p\left( a-1\right)* \exp \right(- em(a)\right)}{\left(1- p\left( a-1\right)\left)*\right( tr(a)+ \exp \left(- i(a)\right)+ p\left( a-1\right)* \exp \Big(- em(a)\right)}\\ {} tr(a)=\frac{ \exp \left(- em(a)\right)- \exp \left(- i(a)\right)}{i(a)- em(a)}\end{array} $$

where

*i(a)*: the incidence in the age interval [a,a + 1)

*RR(a)*: the relative risk on mortality and outflow to institutions in those with the disease in the age interval [a,a + 1) compared to those of similar age without the disease

*m(a)*: the mortality and outflow to institutions in those without the disease in the age interval [a,a + 1)

*em(a)*: the excess mortality (including outflow to institutions) in the age interval [a,a + 1). Excess mortality is defined as the mortality rate in those with the disease minus the mortality rate in those without the disease

*p(a)*: the prevalence at the a^th^ birthday

tr(a): the 1-year transition probability from the state “without the disease” to the state “with disease”

In a second model, we further assumed that a fraction ε of the prevalent cases at the start of each year is misclassified as an incident case in that year. We assume that this fraction ε is the same for all ages. We estimated ε by fitting this model to all data; that is, incidence, prevalence and mortality, using maximum likelihood. Intuitively this implies that when the prevalence as calculated from the incidence and mortality, deviates from the observed prevalence, a fraction ε is sought that makes the deviation as minimal as possible.

In order to use maximum likelihood, we derived the following expected values from this model for the observed entities:

Expected incident cases:

$$ i(a)* p{y}_{without\kern0.5em  disease}(a)+\varepsilon * p(a)* N(a) $$

Expected prevalent cases:

$$ \frac{1}{2}\left( p(a)* N(a)-\varepsilon * p(a)* N(a)\right)+\frac{1}{2}\left( p\left( a+1\right)* N\left( a+1\right)-\varepsilon * p\left( a+1\right)* N\left( a+1\right)\right) $$

Expected number of deaths and institutionalizations in those with the disease

$$ R R(a)* m(a)* p{y}_{with\kern0.5em  disease}(a) $$

Expected number of deaths and institutionalizations in those without the disease

$$ m(a)* p{y}_{without\kern0.5em  disease}(a) $$

where ε is the percentage of prevalent cases that is misclassified as incidence cases by the physician, N(a) is the number in the population at age a and py(a) the number of person years with age a.

With these expected values we can write down the likelihood of the model, assuming a Poisson probability distribution for the number of incident cases and deaths, and a binomial probability distribution for prevalent cases.

## Appendix C

### Life expectancy calculated from mortality data from the General Practice Registration Networks

The figure in this appendix shows the life expectancy for each age group calculated on mortality as registered in the different networks and on mortality data from Statistics Netherlands (Statline). Calculations on mortality data from the RNUH-LEO network, for example, show a life expectancy of 60–65 year old men of almost 30 years, whereas Statistics Netherlands calculates this at about 20 years. For all registration networks life expectancy estimates are too high. The LINH registration performed best, but this might be an artifact, as mortality is LINH is partly derived from periodical checks on whether a patient was still registered, and the average period between check dates used comprise a period that was larger than 1 year, inflating the mortality rates.
